# Supplementary material for: A Likelihood Approach for Real-Time Calibration of Stochastic Compartmental Epidemic Models
Source: PLoS Comput Biol. 2017 Jan 17;13(1):e1005257. doi: 10.1371/journal.pcbi.1005257 (PMC5240920; doi:10.1371/journal.pcbi.1005257)
Supplement: S1 File — (TAR.GZ) [file pcbi.1005257.s014.tar.gz › HSPH_Online-SI-Revision/PF08/n1-extreme/n1-extreme_PF2_summary2.pdf]

peak -8	3w spec	3w cum	5w spec	5w cum	end	3w spec LOG	3w cum LOG	5w spec LOG	5w cum LOG	end LOG
mean	238.2	352.8	69.	140.9	38.7	0.6	0.6	0.5	0.5	0.4
median	162.2	228.	59.7	120.9	34.9	0.6	0.6	0.5	0.5	0.4
quantile .1	58.9	70.3	51.3	54.5	29.4	0.5	0.4	0.5	0.4	0.3
quantile .9	489.8	859.4	96.6	262.3	50.6	0.8	0.9	0.6	0.4	0.5
50	49	45	50	21						

peak -4	3w spec	3w cum	5w spec	5w cum	end	3w spec LOG	3w cum LOG	5w spec LOG	5w cum LOG	end LOG
mean	56.3	53.4	61.	53.4	48.7	0.6	0.5	0.7	0.5	0.5
median	55.3	53.	60.6	52.6	48.3	0.6	0.5	0.7	0.5	0.5
quantile .1	47.8	47.2	53.1	45.2	41.2	0.4	0.3	0.5	0.4	0.4
quantile .9	64.	58.7	69.8	61.3	55.	0.7	0.5	0.8	0.4	0.6
30	37	13	23	11						

peak	3w spec	3w cum	5w spec	5w cum	end	3w spec LOG	3w cum LOG	5w spec LOG	5w cum LOG	end LOG
mean	53.8	53.2	55.8	52.2	49.	0.5	0.4	0.5	0.4	0.4
median	53.1	53.4	56.	52.2	49.4	0.5	0.4	0.5	0.4	0.4
quantile .1	44.1	44.	48.6	44.7	40.7	0.3	0.3	0.3	0.3	0.3
quantile .9	62.8	59.9	62.8	59.	56.	0.6	0.5	0.7	0.3	0.5
23	15	38	17	43						

peak +4	3w spec	3w cum	5w spec	5w cum	end	3w spec LOG	3w cum LOG	5w spec LOG	5w cum LOG	end LOG
mean	79.9	50.9	107.	55.9	74.7	0.4	0.3	0.5	0.3	0.3
median	57.5	47.6	66.6	51.4	63.8	0.4	0.3	0.4	0.3	0.3
quantile .1	45.8	37.7	52.	40.4	46.6	0.2	0.2	0.3	0.2	0.2
quantile .9	135.4	61.6	215.3	69.4	129.3	0.6	0.5	0.7	0.2	0.5
42	43	45	43	46						

peak +8	3w spec	3w cum	5w spec	5w cum	end	3w spec LOG	3w cum LOG	5w spec LOG	5w cum LOG	end LOG
mean	177.1	97.7	812.7	114.4	165.7	0.5	0.4	0.7	0.4	0.5
median	80.4	68.1	123.1	70.6	108.4	0.4	0.3	0.6	0.4	0.4
quantile .1	58.	51.1	69.5	51.5	65.4	0.3	0.2	0.4	0.2	0.3
quantile .9	316.5	190.5	986.	218.5	303.9	0.7	0.5	0.9	0.2	0.6
44	44	46	43	42	{}	{}	{}	{}	{}	{}
